# Modelling the Impact of HIF on Metabolism and the Extracellular Matrix: Consequences for Tumour Growth and Invasion

**DOI:** 10.1007/s11538-024-01391-0

**Published:** 2025-01-03

**Authors:** Kévin Spinicci, Gibin Powathil, Angélique Stéphanou

**Affiliations:** 1 Université Grenoble Alpes, CNRS, UMR 5525, VetAgro Sup, Grenoble INP, TIMC, 38000 Grenoble, France; 2 Department of Mathematics, Swansea University, Swansea, SA1 8EN UK

**Keywords:** Hypoxia inducible factor, Cell migration, Warburg effect, Extra-cellular matrix, Agent-based model

## Abstract

The extracellular matrix (ECM) is a complex structure involved in many biological processes with collagen being the most abundant protein. Density of collagen fibers in the matrix is a factor influencing cell motility and migration speed. In cancer, this affects the ability of cells to migrate and invade distant tissues which is relevant for designing new therapies. Furthermore, increased cancer cell migration and invasion have been observed in hypoxic conditions. Interestingly, it has been revealed that the Hypoxia Inducible Factor (HIF) can not only impact the levels of metabolic genes but several collagen remodeling genes as well. The goal of this paper is to explore the impact of the HIF protein on both the tumour metabolism and the cancer cell migration with a focus on the Warburg effect and collagen remodelling processes. Therefore, we present an agent-based model (ABM) of tumour growth combining genetic regulations with metabolic and collagen-related processes involved in HIF pathways. Cancer cell migration is influenced by the extra-cellular collagen through a biphasic response dependant on collagen density. Results of the model showed that extra-cellular collagen within the tumour was mainly influenced by the local cellular density while collagen also influenced the shape of the tumour. In our simulations, proliferation was reduced with higher extra-cellular collagen levels or with lower oxygen levels but reached a maximum in the absence of cell-cell adhesion. Interestingly, combining lower levels of oxygen with higher levels of collagen further reduced the proliferation of the tumour. Since HIF impacts the metabolism and may affect the appearance of the Warburg Effect, we investigated whether different collagen conditions could lead to the adoption of the Warburg phenotype. We found that this was not the case, results suggested that adoption of the Warburg phenotype seemed mainly controlled by inhibition of oxidative metabolism by HIF combined with oscillations of oxygen.

## Introduction

The extracellular matrix (ECM) is a complex structure that can dictate the behaviour of the cell by influencing its proliferation, growth, migration and apoptosis. While its composition can greatly vary between tissues, it contains 300 types of proteins, water, polysaccharides and proteoglycans. ECM proteins can influence tissue homeostasis, organ development, inflammation and diseases. Most of these proteins are fibrous proteins like collagen, elastin, fibronectin and laminin Gonçalves and Garcia-Aznar (2021). Cell migration direction and speed can be influenced by the ECM stiffness, *i. e.* its resistance to deformation in response to applied forces. Motility and invasion, ability to proliferate and colonize other tissues are among the essential hallmarks of metastatic cells Lah et al. (2020).

These hallmarks are influenced by collagen: the most abundant fibrous protein. Fibroblasts represent the main source of collagen and are responsible for both the organization and the alignment of collagen fibres. Mutations of collagen have been associated with diseases like osteoporosis. The collagen family includes 28 different collagen types classified into seven different categories Theocharis et al. (2016). The synthesis of collagen is a complex process that involves several post-translational modifications and the assembly of pro-collagen chain, secretion and processing to form the collagen fibres network Hong et al. (2004). Literature has demonstrated that three families of genes that are involved in the collagen modification of the ECM: the the prolyl 4-hydroxylase (P4H), matrix metalloproteinases (MMPs) and the lysyl oxidase (LOX) Gilkes et al. (2014); Wong et al. (2011); Germain et al. (2010); Myllyharju and Schipani (2010). Cell-fibres interactions represent a major component for cell migration as cells are slower in stiffer matrix, yet they tend to migrate to stiff areas of the matrix Schlüter et al. (2012). In cancer, enhanced deposition of collagen is the most recognized alteration of tumorous tissues. Cancer cells are able to stimulate the synthesis of ECM proteins through cancer associated fibroblast (CAF) recruitment. Increased deposition of collagen has been observed in hypoxic areas of the tumour Gilkes et al. (2014).

Hypoxia is a major phenomenon observed in cancer as the median observed tumour oxygen is 2 % McKeown (2014). In glioblastoma, the inconsistent oxygenation due to the altered blood vessels causes hypoxia, increased acidity and necrosis Monteiro et al. (2017). The increased resistance to radiotherapy of hypoxic cells was attributed to the Hypoxia Inducible Factor (HIF) Leung et al. (2017). The HIF protein is the main actor involved in the cellular response to hypoxia. The most known isoform HIF1-$\upalpha$ acts as a transcription factor that can up-regulate the transcriptional activity of target genes called hypoxia response elements (HRE). 98 genes have been identified as HRE Slemc and Kunej (2016). They are involved in various biological functions such as cell proliferation, survival, apoptosis, erythropoiesis and angiogenesis Lee et al. (2004). HIF is mainly regulated at the protein level by oxygen-dependant degradation mechanisms Masoud et al. (2015). It can affect the levels of the two metabolic genes lactate dehydrogenase (LDH) and pyruvate dehydrogenase (PDH) Bedessem (2015); Lee et al. (2004). Hence, HIF may be a regulator of the Warburg Effect which is characterized by an increased production of lactate (even in normoxia) induced by an increased usage of the glycolytic pathways. HIF1-$\upalpha$ has been recognized as a master regulator of the epithelial to mesenchymal transition (EMT), invasion and metastasis in breast cancer D’Ignazio et al. (2017). Exposure of cells to hypoxia in glioblastoma elevated the levels of EMT associated genes Xu et al. (2015). Interestingly, high matrix stiffness can inhibit angiogenesis Germain et al. (2010).

Because metastasis is a major factor hindering therapy in cancer, cell migration has been studied through mathematical modeling which included different processes such as collagen degradation, cell-cell adhesion or the effect of a heterogeneous matrix Schlüter et al. (2015); Anderson (2005); Gonçalves and Garcia-Aznar (2021); Deakin and Chaplain (2013); Rubenstein and Kaufman (2008). It should be noted that the processes mentioned are occuring at the early stage of the metastatic process while, in later stages, the cell escapes its original tissue and starts invading surrounding ones. Among the models of cellular migration mentioned above, none investigated the effect of HIF on migration despite hypoxia being a common factor in cancer which promotes invasion. While HIF has been mathematically described, it was mainly to understand its biology (*e. g.* its regulation by oxygen-dependant mechanisms) using ordinary differential equations (ODEs) based systems (see Cavadas et al. (2013); Bedessem and Stéphanou (2014a, 2014b); Jia et al. (2019)).

In a previous study, we investigated the effect of HIF on the Warburg Effect through its impact on the LDH and PDH genes in the case of glioblastoma. We have shown that the Warburg phenotype could be triggered when the oxygen-dependant degradation of HIF was reduced or when oxygen diffusion varied through the tumour growth Spinicci et al. (2022).

While the ECM in the brain lacks rigid structure such as fibrous protein Vollmann-Zwerenz et al. (2020), it has been observed that the matrix in glioblastoma has a higher collagen content than in a healthy brain Gonçalves and Garcia-Aznar (2021). In progressive glioma, the brain matrix becomes more disorganized and stiffer with the growing malignancy of the tumour Lah et al. (2020). Furthermore, it has been shown that glioblastoma cells are able to produce collagen fibres like fibroblasts Kaufman et al (2005); Pranita et al. (2019). Hence in this paper, we present a mathematical model of the impact of HIF on cellular migration through its effect on collagen remodeling processes in the case of glioblastoma. We developed an agent-based model (ABM) to characterize the interaction between the cell and the ECM at the cellular level. Modification of the collagen content is influenced by the level of HIF. The model describes the impact of collagen on the cell migration speed, to reflect the differences between tumour growth in different collagen conditions.

## Materials and Methods

### Existing Model

The model presented in this paper is based on our precedent work on the study of the impact of HIF on the metabolic key genes LDH and PDH Spinicci et al. (2022). The levels of LDH and PDH enzymes control the usage of aerobic and anaerobic pathways to produce the cell’s Adenosine Triphosphate (ATP). The paper aimed to study how different levels of HIF can lead to the appearance of the Warburg effect during tumour growth in different conditions. The model combined a gene regulatory network with equations for metabolism influenced by the levels of LDH and PDH described by ODE:

1 $$\begin{aligned} \frac{dh}{dt}&= \alpha _h \ \beta _h - d_h \ S(O, s_{O \rightarrow h}, \gamma _{O \rightarrow h}) \ h , \end{aligned}$$

2 $$\begin{aligned} \frac{dl}{dt}&= \alpha _l \ S(h, s_{h \rightarrow l}, \gamma _{h \rightarrow l}) - d_l \ l , \end{aligned}$$

3 $$\begin{aligned} \frac{dk}{dt}&= \alpha _k \ S(h, s_{h \rightarrow k}, \gamma _{h \rightarrow k}) - d_k \ k , \end{aligned}$$

4 $$\begin{aligned} \frac{dq}{dt}&= \alpha _q \ S(k, s_{k \rightarrow q}, \gamma _{k \rightarrow q}) - d_q \ q , \end{aligned}$$

with *h*, *l*, *k* and *q* the levels of HIF, LDH, PDK and PDH. Gene regulations were simulated using the Shifted-Hill function Li and Wang (2020), a non-linear function to depict gene up- and down- regulation depending on a regulating gene. The Shifted-Hill function consists mainly of the summation of two Hill terms:

5 $$\begin{aligned} S(Y, s, \gamma ) = \frac{ s^n }{ s^n + Y^n } + \gamma \frac{ Y^n }{ s^n + Y^n } . \end{aligned}$$

Here *n* is the Hill coefficient, *s* is the level of the regulating protein *Y* at which the regulation is half-maximum, $\gamma$ is the regulating strength of *Y* on its target with $\gamma \in \mathbb {R}_{+}$. If $\gamma > 1$ then $S(Y, s, \gamma )$ indicates an up-regulation, if $\gamma < 1$ then $S(Y, s, \gamma )$ indicates a down-regulation.

Metabolism is mainly simulated using Michaelis-Menten function Robertson-Tessi et al. (2015). The model described extra-cellular quantities of oxygen, glucose and H^+^ through partial differential equations (PDEs):

6 $$\begin{aligned} \frac{ \partial O }{ \partial t}&= D_O \nabla ^2 O - \sum _{N}^{i=1} f_{O}^{i} , \end{aligned}$$

7 $$\begin{aligned} \frac{ \partial G }{ \partial t}&= D_G \nabla ^2 G - \sum _{N}^{i=1} f_{G}^{i} , \end{aligned}$$

8 $$\begin{aligned} \frac{ \partial H }{ \partial t}&= D_H \nabla ^2 H + \sum _{N}^{i=1} f_{H}^{i} . \end{aligned}$$

With $D_O$, $D_G$ and $D_H$ the diffusion coefficients of the different diffusibles. $f_O$ and $f_G$ are functions describing the consumption of oxygen and glucose, respectively, while $f_H$ determines the secretion of protons. Here, we sum the consumption and secretion $f^i$ for each cell *i* to *N* in the current voxel.

**Generation of ATP at each timestep**:

9 $$\begin{aligned} \frac{ dA }{ dt } = f_A . \end{aligned}$$

See (14) for more details on ATP generation.

**Oxygen** (*O*) **consumption**:

10 $$\begin{aligned} f_O&= \Psi _O V_O \frac{O}{K_O + O} , \end{aligned}$$

11 $$\begin{aligned} \Psi _O&= \frac{ \Phi _O - \phi _O }{ 1 + \exp { \bigl (\ -\lambda _q (q - q_0)\ \bigr ) } } + \phi _O . \end{aligned}$$

$V_O$ is the maximal consumption rate of oxygen and $K_O$ is the concentration of oxygen at which the consumption is half-maximal (Michaelis-Menten constant of oxygen). $\Psi _O$ is a logistic function to tune the consumption of oxygen depending on the level of the gene PDH. $\Phi _O$ and $\phi _O$ represent the maximum and minimum values, $\lambda _q$ is the steepness of the curve, *q* is the level of PDH and $q_0$ is the level at which the effect of PDH is half-maximal (midpoint of the logistic curve, similar to the Michaelis-Menten constant).

**Glucose** (*G*) **consumption**:

12 $$\begin{aligned} f_G&= \left( \frac{ \Psi _G A_0 }{ 2 } - \frac{ 29 f_O }{ 10 } \right) \frac{G}{K_G + G} , \end{aligned}$$

13 $$\begin{aligned} \Psi _G&= \frac{ \Phi _G - \phi _G }{ 1 + \exp { \bigl (\ -\lambda _l (l - l_0)\ \bigr ) } } + \phi _G . \end{aligned}$$

$A_0$ is the target ATP demand the cell needs to meet, $K_G$ is the concentration of glucose at which the consumption is half-maximal. Glucose consumption is influenced through the term $\Psi _G$ which represents the effect of the LDH gene on glucose consumption. $\Phi _G$ and $\phi _G$ represent the maximum and minimum values, $\lambda _l$ is the steepness of the curve, *l* is the level of LDH and $l_0$ is the level at which the effect of LDH is half-maximal.

**ATP** (*A*) **production**:

14 $$\begin{aligned} f_A = 2 f_G + \frac{29}{5} f_O . \end{aligned}$$

Here, levels of ATP generated are computed from the consumption of glucose and oxygens multiplied by their respective stoichiometric coefficients.

**H**^+^ (*H*) **secretion**:

15 $$\begin{aligned} f_H = 2 f_G \ k_H . \end{aligned}$$

$k_H$ is a proton-buffering term.

Accurate depiction of oxygen diffusion would lead to a better representation of HIF levels within the tumour. Thus we developed an ABM using the PhysiCell software Ghaffarizadeh et al. (2018), a C++ software designed to run simulations of tumour growth for a large population of cells. The software displays good performance with low memory footprint and fast simulation thanks to parallelized computation. Cell state in the model was updated at each $dt_{phenotype}$ using the level of ATP generated at each timestep and the local extracellular pH. If levels of ATP or pH generated were too low, the cell dies. If pH was sufficient and the levels of ATP generated were high enough for the cell to survive but not enough for division, then the cell enters quiescience. Lastly, if levels of ATP generated were high and pH was not too low, then the cell is allowed to divide with no conditions on the cell neighborhood.

### Effect of HIF on the Nature and Content of Collagen

Collagen is a molecule that undergo complex post-translational modification which modifies its properties as well as the properties of the resulting ECM Gilkes et al. (2014). While ECM in the brain is known to lack rigid structures Vollmann-Zwerenz et al. (2020), brain in glioblastoma has a higher collagen content Lah et al. (2020) with a stiffer and more disorganized matrix than healthy brain Xiong et al. (2018). Hence, in this paper, we focus on the action of three different families of proteins namely of the P4Hs family, MMPs family and LOXs family impacting the formation, degradation and cross-linking of collagen fibres Germain et al. (2010); Wong et al. (2011); Myllyharju and Schipani (2010). These families were selected due to their up-regulation by HIF.

Proline residues of procollagen are hydroxylated by members of the P4H which is an essential step for the proper folding of collagen chains enabling them to form a triple helical structure, promoting the formation of collagen fibres. P4HA1 and P4HA2, two P4H isoforms, are found to be up-regulated by HIF1-$\upalpha$Monteiro et al. (2017); Zhu and Jin (2020); Xiong et al. (2018); Brereton et al. (2022); Lin et al. (2021). Yet we only modelled the P4HA1 isoform as no differences between the two proteins were documented.

Cancer cell migration is favoured by matrix degradation creating available space for the cell to migrate into. Many molecules can degrade collagen such as the “A disintegrin and metalloproteinases (ADAM)” family and cathepsins Guo and Giancotti (2004); Amar et al. (2017); Musumeci et al. (2015); Tam et al. (2004). Yet, the main collagen degradation activity is attributed to a family of Zn^2+^ dependent edopeptidases called matrix metalloproteinases (MMPs). Various MMP isoforms have been identified, among them MMP2/MMP9 and MT1-MMP (also called MMP14) have been observed to be up-regulated by HIF Gilkes et al. (2014); Brereton et al. (2022); Aktar et al. (2009); Petrella et al. (2005); Wan et al. (2011); Sakamoto and Seiki (2017); Unwith et al. (2015). Despite that MMP2 and MMP9 are considered to be oncogenes Ramachandran et al. (2017); Varani et al. (2000); Musumeci et al. (2015), only MT1-MMP is included in the model, a choice motivated by several reasons. The activity of several MMPs is redundant and compensatory, hence modelling several MMPs would not give more accurate results and would increase the complexity of the model. In addition, MMP2 activation is dependent on the tissue inhibitors of MMP (TIMP)-2 factor and MT1-MMP Itoh and Seiki (2006); Hoshino et al. (2012); Karagiannis et al. (2004), suggesting that MT1-MMP would be a limiting factor for MMP2 activation. Furthermore, MT1-MMP could promote cellular invasiveness but not soluble MMPs Itoh and Seiki (2006). Lastly, MMP2 collagenolytic activity seems to be ambiguous. MMP2 may be important for the clearance of degraded and denatured collagen partially cleaved by MT1-MMP beforehand Amar et al. (2017); Tam et al. (2004).

Cross-linking of collagen fibrils by LOX stabilizes the collagen fibrils which gives collagen its tensile strength and mechanical stability Hong et al. (2004). HIF is able to up-regulate three members of the LOX family, here we include only the activity of the LOX gene as it seems that cross-linking activity is most often attributed to this isoform.

**Fig. 1:**
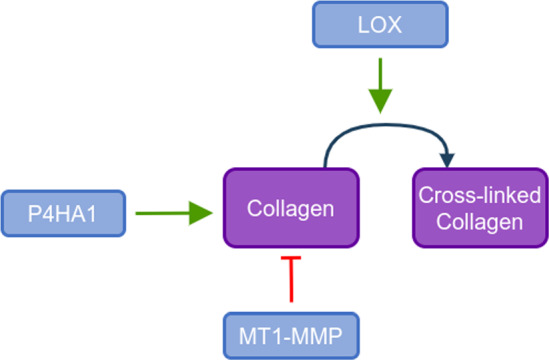
Secretion, degradation and cross-linking of collagen mediated by P4H1A, MT1-MMP and LOX

The effects of the different genes on collagen described here are summarized in Fig. 1.

Gene levels are described using the following set of ODE:

16 $$\begin{aligned} \frac{dp}{dt}&= \alpha _p \ S(h, s_{h \rightarrow p}, \gamma _{h \rightarrow p}) - d_p \ p , \end{aligned}$$

17 $$\begin{aligned} \frac{dm}{dt}&= \alpha _m \ S(h, s_{h \rightarrow m}, \gamma _{h \rightarrow m}) - d_m \ m , \end{aligned}$$

18 $$\begin{aligned} \frac{dr}{dt}&= \alpha _r \ S(h, s_{h \rightarrow r}, \gamma _{h \rightarrow r}) - d_r \ r , \end{aligned}$$

where *p*, *m* and *r* describe the levels of P4HA1, MT1-MMP and LOX. Here, $\alpha _x$ is the production rate of the protein *x* and $d_x$ is the degradation rate of the protein *x*. Up-regulation of P4HA1, MT1-MMP and LOX by HIF are described using the Shifted-Hill equation (Equation 5).

Parameters for the Shifted-Hill equation were estimated using glioblastoma bulk tumour RNA-Seq data from the TCGA-GBM project available on the The Cancer Genome Atlas (TCGA) database. Parameters were normalized as follows:

1. Normalization of the count data using the |normTransform| function from the DESeq2 R package Love et al. (2014) (version 1.40).
2. Interpolation of normalized values:
  $$\begin{aligned} \hat{x}: f(x) = \frac{ x - x_{min} }{ x_{max} - x_{min} }, \text { with } \hat{x} \in [0,1]. \end{aligned}$$
3. The Shifted-Hill function is the summation of two Hill terms: an activating term and an inhibiting one. Only the activating term is effective when $\gamma > 1$ (when *Y* up-regulates *X*), while only the inhibiting term is effective when $\gamma < 1$ (when *Y* down-regulates *X*). Therefore, we use this property to fit the parameter *s* on different functions depending on the value of $\gamma$:
  $$\begin{aligned} h(Y)&= \frac{Y^n}{s^n + Y^n}, \text { if } \gamma >1 \text {(up-regulation)} \\ h(Y)&= \frac{s^n}{s^n + Y^n}, \text { if } \gamma <1 \text {(down-regulation)} \end{aligned}$$
  *Y* is the regulating gene, *n* is the Hill coefficient, *s* is the concentration of *Y* at which the effect on *X* is half-maximal. The Hill coefficient is fixed to 4, the value used in the model.
4. The parameter *s* is then rescaled to the original dimensions of the data before Interpolation.
5. The $\gamma$ parameter is estimated using the fold-change of expression in the data:
  $$\begin{aligned} \text{ fold-change }&= \frac{ x_{max} }{ x_{min} }, \text{ if } \text{ the } \text{ gene } \text{ X } \text{ is } \text{ up-regulated }, \\ \text{ fold-change }&= \frac{ x_{min} }{ x_{max} }, \text{ if } \text{ the } \text{ gene } \text{ X } \text{ is } \text{ down-regulated }. \end{aligned}$$

Initial protein level were set to 1.0 in every simulations ran. Parameters for genetic regulation are summarised in table 1.

**Table 1:** Parameters for genetic regulations in the model of the impact of HIF on the cellular invasion

Parameter	Value	Dimension	Parameter	Value	Dimension
$\alpha _h, \alpha _l, \alpha _k, \alpha _q, \alpha _p, \alpha _m, \alpha _r$	0.005	$min^{-1}$	$d_h, d_l, d_k, d_q, d_p, d_m, d_r$	0.005	$min^{-1}$
$s_{O \rightarrow h}$	0.02085	mmol/L	$s_{h \rightarrow l}$	13.04	–
$s_{h \rightarrow k}$	13.34	–	$s_{k \rightarrow q}$	9.94	–
$s_{h \rightarrow p}$	13.68	–	$s_{k \rightarrow m}$	13.25	–
$s_{h \rightarrow r}$	13.44	–			
$\gamma _{O \rightarrow h}$	25.01	–	$\gamma _{h \rightarrow l}$	16.06	–
$\gamma _{h \rightarrow k}$	17.87	–	$\gamma _{k \rightarrow q}$	0.78	–
$\gamma _{h \rightarrow p}$	16.87	–	$\gamma _{k \rightarrow m}$	20.43	–
$\gamma _{h \rightarrow r}$	25.46	–			
$\beta _h$	25.01	–	*n*	4	–

The symbol "-" stands for dimensionless

### Implementation of the ECM and Collagen Modifying Processes

The PhysiCell software simulates the microenvironment of the cell as a 2D square-grid lattice. Each element of the grid is called a voxel, extracellular quantities vary between each voxel. The ECM is not explicitly modeled in PhysiCell, instead we describe the extra-cellular collagen as a substrate that does not diffuse nor decay. Extensive characterization of collagen through complete mechanical interaction or modeling of the fibres is out of the scope of this study. This study aims to characterize instead the variations of collagen content caused by HIF. Modelling collagen as a substrate still retains the ability to describe different states of collagen namely “collagen” and “cross-linked collagen”. Cross-linked collagen participates in the matrix stiffness yet it is resistant to degradation by MMP Amar et al. (2017); Tam et al. (2004).

Collagen fibres are composed of several procollagen $\upalpha$ chains coded by the COL genes (COL1A1, COL1A3, etc). These chains undergo post-translational modification by P4Hs proteins which favour the assembling of procollagen into collagen fibres. We hypothesize that the formation of collagen fibres is dependent upon the activity of P4HA1, since unprocessed procollagen does not form any collagen fibres and consequently, does not participate in the collagen content of the ECM. As for secretion, collagen degradation and cross-linking are influenced by the levels of MT1-MMP and LOX. Compared to the secretion of collagen, processes of degradation and cross-linking require collagen fibres as a substrate. Consequently, degradation/cross-linking should not only rise as enzyme levels increase but fall as collagen quantity decreases as well. Collagen degradation and cross-linking are modeled using Michaelis-Menten dynamics. MT1-MMP activity has already been described using Michaelis-Menten dynamics in the literature Hoshino et al. (2012). The resulting functions to describe collagen dynamics are:

19 $$\begin{aligned} f_C&= \beta _p p - \beta _m m \frac{C}{K_m + C} - \beta _r r \frac{C}{K_r + C} , \end{aligned}$$

20 $$\begin{aligned} f_{C_r}&= \beta _r r \frac{C}{K_r + C} , \end{aligned}$$

where $f_C$ and $f_{C_r}$ describe the variations of collagen *C* and cross-linked collagen $C_r$ in the environment. Levels of P4HA1, MT1-MMP and LOX are described by the variables *p*, *m* and *r*. Parameters $\beta _p$, $\beta _m$ and $\beta _r$ are the base rate of collagen secretion, degradation by *m* and cross-linking by *r*. $K_m$ is the level of *m* at which the degradation rate of collagen is half-maximal, and $K_r$ is the level of *r* at which the cross-linking rate is half-maximal.

While degradation of the ECM is essential for cellular migration, ECM acts as an important scaffolding for migration, hence its degradation must be localized Itoh and Seiki (2006). MT1-MMP is a membrane-bound protein usually located on the invadopodia or lamellipodia: the migration front of the cell Hoshino et al. (2012); Itoh and Seiki (2006). Consequently, the distance of MT1-MMP mediated degradation should be limited by the cell in the model. The cell will only degrade the collagen of the voxel it is currently inside. For simplicity of implementation, LOX-mediated cross-linking will follow the same rule. PhysiCell does not have built-in interaction between substances; which means that it does not describe any interactions of the type “the **Substance A** influences the concentration of a **Susbtance B**”. Hence, interactions between extracellular LOX and collagen would need to be implemented manually. The unit to quantify the density of collagen and cross-linked collagen is mg/mL, the unit the most often reported when conducting gel experiments. The extracellular density of collagen is described by the PDEs:

21 $$\begin{aligned} \frac{ \partial C }{ \partial t}&= \sum _{N}^{i=1} f_{C}^{i} , \end{aligned}$$

22 $$\begin{aligned} \frac{ \partial C_r }{ \partial t}&= \sum _{N}^{i=1} f_{C_r}^{i} . \end{aligned}$$

Here $f_{C}^{i}$ and $f_{C_r}^{i}$ are the quantity of collagen degraded and the quantity of collagen cross-linked by the cell *i*, *N* is the total number of cell in the current voxel.

Let $x_0$ and $y_0$ the lower boundary of the domain in *x* and *y*, $x_L$ and $y_L$ the upper boundary in *x* and *y*. Simulations were run with the following initial conditions:

- Oxygen: $O(x,y,0) = 0.056$ mmol/L (normoxia or 5 % O_2_ ) McKeown (2014).
- Glucose: $G(x,y,0) = 5.0$ mmol/L (serum glucose) Patel et al. (2001).
- H^+^ : $$H^+(x,y,0) = 3.98 \times 10^{-5}$$ mmol/L (pH 7.4).
- Collagen: $C(x,y,0) = 2.5$ mg/mL (optimal collagen density for growth Schor et al. (1982)).
- Cross-linked Collagen: $C_r(x,y,0) = 0.0$ mg/mL.

And the following Dirichlet-Boundary conditions:

- Oxygen: $$O(x_0,y,t) = O(x_L,y,t) = O(x,y_0,t) = O(x,y_L,t) =$$ 0.056 mmol/L.
- Glucose: $$G(x_0,y,t) = G(x_L,y,t) = G(x,y_0,t) = G(x,y_L,t) = 5.0$$ mmol/L.
- H^+^ : $$H^+(x_0,y,t) = H^+(x_L,y,t) = H^+(x,y_0,t) = H^+(x,y_L,t) = 3.98 \times 10^{-5}$$ mmol/L (pH 7.4).
- Collagen and Cross-linked collagen: No boundary conditions as they do not diffuse.

**Table 2:** Parameters for metabolism

Parameter	Value	Unit	Description
* Oxygen*			
$V_O$	0.01875	*mmol*/*L*/*min*	Maximal oxygen consumption rate
$K_O$	0.0075	*mmol*/*L*	Michaelis-Menten constant of oxygen consumption
$\Phi _O$	1	–	Maximum value of $\Psi _O$
$\phi _O$	0	–	Minimum value of $\Psi _O$
$\lambda _q$	15	–	Steepness of $\Psi _O$
$q_0$	0.575	–	Midpoint of $\Psi _O$
* Glucose*			
$K_G$	0.04	*mmol*/*L*	Michaelis-Menten constant of glucose consumption
$A_0$	0.10875	*mmol*/*L*/*min*	Target ATP level
$\Phi _G$	50	–	Maximum value of $\Psi _G$
$\phi _G$	1	–	Minimum value of $\Psi _G$
$\lambda _l$	4	–	Steepness of $\Psi _G$
$l_0$	8.03	–	Midpoint of $\Psi _G$
*H*^+^			
$K_H$	$2.5 \cdot 10^{-4}$	–	Proton buffering term
* collagen*			
$\beta _p$	$4.04 \times 10^{-6}$	*mg*/*mL*/*min*	Base collagen secretion rate by P4HA1
$\beta _m$	$2.16 \times 10^{-5}$	*mg*/*mL*/*min*	Base collagen degradation rate by MT1-MMP
$K_m$	$8.7 \times 10^{-11}$	*mg*/*mL*	Michaelis-Menten constant of MT1-MMP
$\beta _r$	$2.91 \times 10^{-10}$	*mg*/*mL*/*min*	Base collagen cross-linking rate by LOX
$K_r$	$6.75 \times 10^{-9}$	*mg*/*mL*	Michaelis-Menten constant of LOX
* Diffusion*			
$D_O$	109, 200	$\mu m^2/min$	Oxygen diffusion coefficient
$D_G$	30, 000	$\mu m^2/min$	Glucose diffusion coefficient
$D_{H^+}$	27, 0000	$\mu m^2/min$	H^+^ diffusion coefficient

The symbol "-" stands for dimensionless

The list of parameters for the model can be found in table 2.

### Integration of Cell Migration

**Fig. 2:**
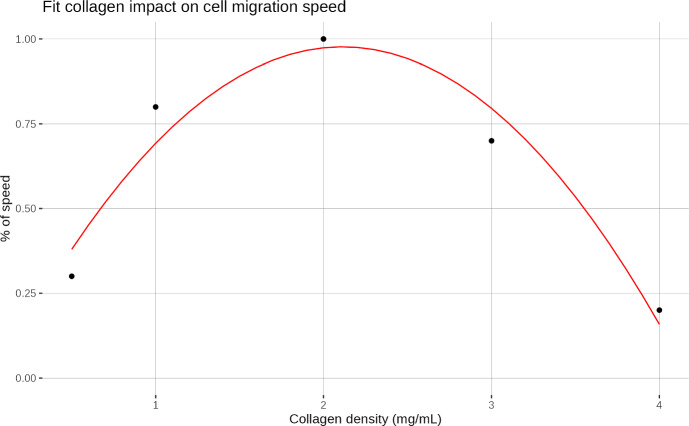
Result of the fit of a second-degree polynomial on the data from Schor et *al* Schor et al. (1982)

ECM collagen content is an important factor that influences both the ability of the cell to migrate and its speed Pfisterer et al. (2021). Experiments of cellular migration on collagen gels with different properties show a biphasic effect of collagen on the migration speed of the cell Schor et al. (1982); Lang et al. (2015). Studies report that the cellular migration speed in different collagen densities is a bell-shaped curve. At the beginning, the speed of the cell increases with higher collagen densities until a maximum is reached, which is reported to be 1.2 or 2.0 mg/mL of collagen Schor et al. (1982); Lang et al. (2015). After that maxima, the cell migration speed decreases as collagen densities continue to rise. In addition, it has been observed that cells are less invasive in a softer matrix (0.5 mg/mL of collagen) Kaufman et al (2005). Following the observation made by Schor et *al* Schor et al. (1982), we only allow cellular migration for collagen densities between 0.5 and 4.0 mg/mL. We fitted a second-degree polynomial using their data with values in [0, 1] to modify the speed of the cell from 0 up to a maximum. Data were normalized between 0 and 1 prior to fitting. Figure 2 shows the result of the fit and the impact of collagen.

The speed of the cell is represented by the following piecewise function :

23 $$\begin{aligned} \nu = {\left\{ \begin{array}{ll} 0 & \text {if } C_t \notin [0.5, 4.0] \text { mg/mL,} \\ \nu _{0} (-0.23 x^2 + 0.971 x - 0.048) & \text {if } C_t \in [0.5, 4.0] \text { mg/mL,} \\ \end{array}\right. } \end{aligned}$$

where $\nu _0$ is the base speed of the cell and $C_t$ the total density of collagen ($C_t = C + C_r$). Here $\nu _0$ is equal to 0.8 $\upmu$ m/min Kaufman et al (2005).

## Results

### Extra-cellular Collagen Within the Tumour is Impacted by the Local Cellular Density

**Fig. 3:**
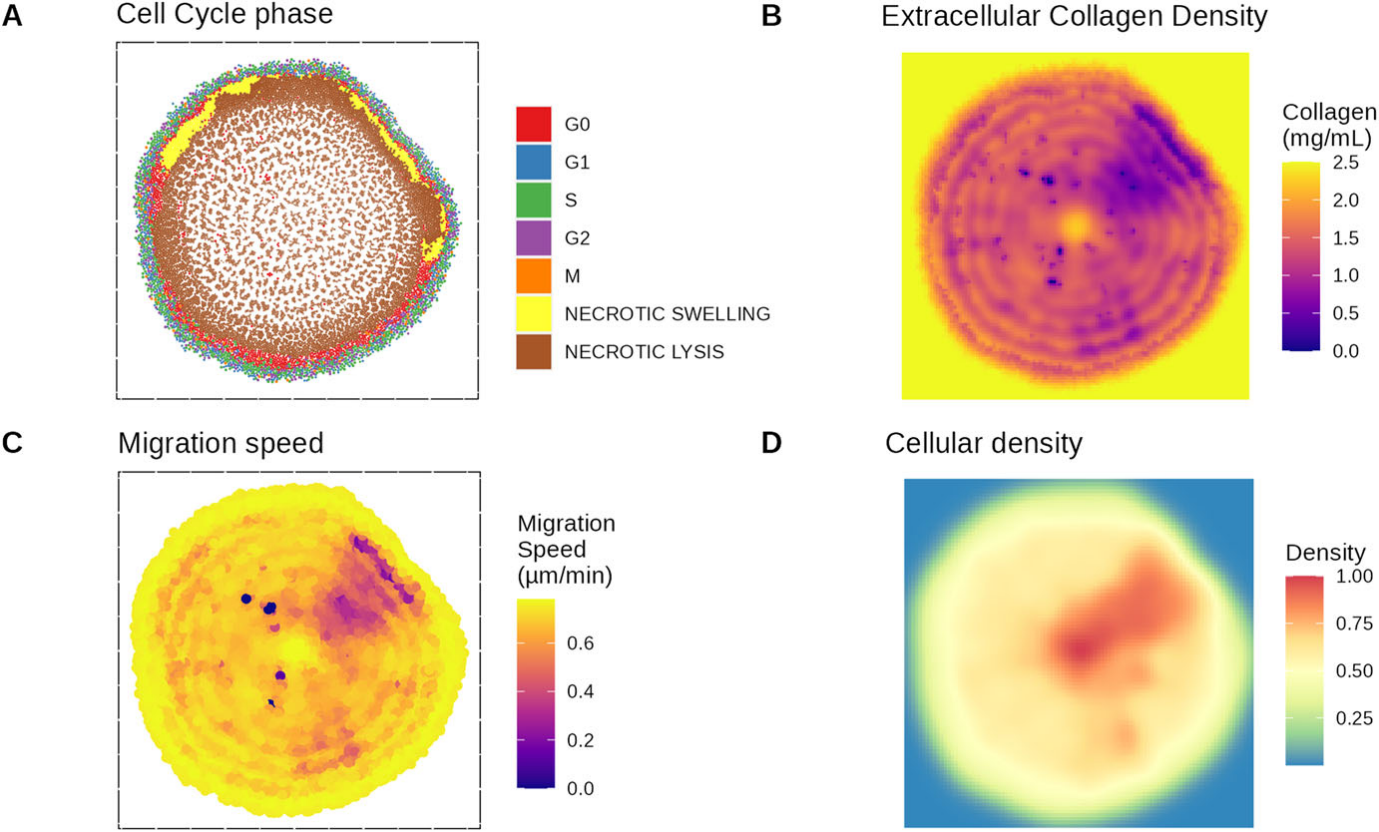
Tumour after 14 days grew in normoxia with the default initial conditions and parameters. The figure shows the (A) cell-cycle phase, (B) extracellular collagen, (C) migration speed and (D) normalized cellular density within the tumour

A simulation was run with default parameters and initial conditions: homogeneous and normoxic oxygen conditions with a homogeneous collagen matrix at a concentration optimal for growth. Figure 3 shows the resulting tumour along with the extracellular collagen and the cellular density within the tumour. After 14 days of growth, the center of the tumour is mainly composed of necrotic cells while the periphery is composed of proliferative cells. A thin layer of quiescent cells can be observed between the necrotic cells and the proliferative cells. This layer is thicker at the bottom of the tumour compared to the top. On the opposite, a group of necrotic swelling cells is present on the upper part of the tumour.

Some cells tend to detach from the tumour’s main body and move slightly away, although it is not significant enough to call those cells “invasive”. This observation is similar to the results from Rubenstein et *al* Rubenstein and Kaufman (2008) with their model of cell migration based on a cellular Potts model. Because collagen is an important aspect of the model, it appears essential to investigate how the tumour impacts extracellular collagen density. Collagen density within the tumour ranges from 0 to 2.0 mg/mL. The highest density of collagen inside the tumour is found at its centre where tumour growth started. At the beginning of the simulation, cells are in normoxia and may not alter much the collagen content. Because cells at the centre of the tumour are the first to die due to harsh conditions, they may not had the time to alter significantly their environment.

Interestingly, the pattern of extracellular collagen is similar to the pattern of the local cellular density and the cellular migration speed. Areas with higher local cellular density display decreased extracellular collagen levels and cellular migration speed. This suggests that a higher number of cells leads to an increased degradation of collagen which affects the cellular migration speed. Consequently, cellular migration speed decreases when the cellular density rises. The reason why cellular density locally rises is unknown. However, it could be explained by the asynchronicity of the cell cycle. The cell cycle phase is randomized at the beginning of the simulation to replicate more accurately the growth that could be observed *in vivo*. Thus, a few cells may divide more early than others cells as they are in a later stage of the cell cycle which increases the local cellular density.

### Tumour Proliferation is Impacted by Extracellular Collagen, Oxygen and Cellular Adhesion Forces

To understand the impact of environmental collagen and oxygen on tumour growth, different environmental settings were tested:

- Bi-Gel: The microenvironment is split into two collagen densities like a collagen gel with a soft part (2.5 mg/mL) and a stiff one (5.0 mg/mL) to assess the impact two densities may have on the tumour.
- Lower/Upper Oxygen: Oxygen only diffuses at the top and the bottom of the domain to determine how cells with lower access to oxygen respond.
- Bi-Gel + Lower/Upper Oxygen: Combination of the Bi-Gel condition and the Lower/Upper Oxygen settings to observe the impact of both conditions.
- Complex matrix: Collagen density is randomly generated following a uniform distribution between 0.5 and 4.0 mg/mL to simulate a heterogeneous environment comparable to *in vivo* conditions.
- No Cell-Cell adhesion: In that case, cell-cell adhesion forces are null as migrating cells are known to have reduced cell-cell adhesion and increased cell-matrix interactions. Cellular adhesion forces can be controlled through a parameter defined in the PhysiCell software.

**Fig. 4:**
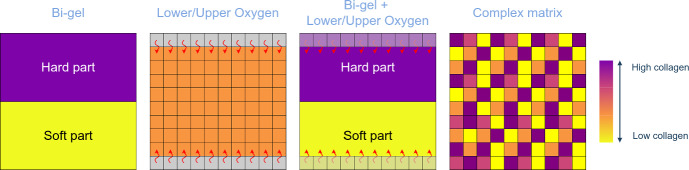
Different environmental settings represented graphically: Bi-Gel with a stiff part and soft part; Lower/Upper oxygen with diffusion at the top and the bottom of the domain; Combination of Bi-Gel and Lower/Upper oxygen; Complex matrix with randomly initiated values of collagen

**Fig. 5:**
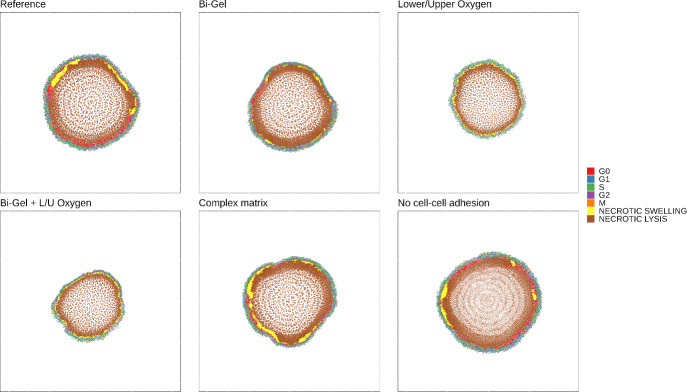
Tumour growth in different conditions with the indication of cell cycle

Figure 4 graphically describes the different collagen and oxygen settings used. Tumour growth after 14 days in these different conditions is shown in Fig. 5. Like in the reference, the tumour in every simulation is composed of a necrotic core with a surrounding layer of proliferative cells. Interestingly, cells tend to pack together when dying in the necrotic core of the majority of simulations run except in the simulation with no cell-cell adhesion. With no cellular adhesion, the cell spatial distribution shows the same wave pattern as extracellular collagen in Fig. 3, similar to the results generated by the model from Anderson Anderson (2005). The tumour has a “roundish” shape in the reference, it has a “pear” shape in the Bi-Gel and seems to form some branching in the complex matrix. Formation of “branching-like” structure is also observed in the model from Anderson Anderson (2005) yet in the latter, these structures are more pronounced than in our results. In Anderson’s model, the tumour is grown for a longer time than in this study which may explain why the results are different. The results obtained in different collagen conditions suggest that extracellular collagen only is able to shape the tumour during growth. This may be explained by the differences in migration speed among the cells caused by different collagen conditions.

**Fig. 6:**
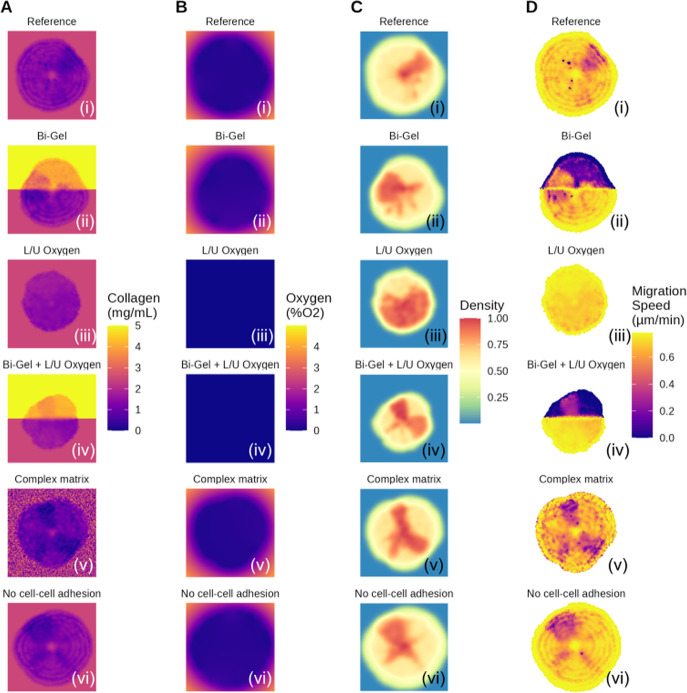
Comparison of (A) the extracellular collagen, (B) extracellular oxygen, (C) the cellular density and (D) the cell migration speed in the different settings studied

Figure 6 shows the extracellular collagen, extracellular oxygen, cellular density and the cell migration speed in the different simulations after 14 days of growth. Similar to the observations with the reference simulation (Fig. 3), the matching pattern indicates a possible link. As can be observed, regions with lower migration speed values colocate with hollow areas where the “branches” of the tumour are forming. As a result, areas with lower collagen levels result in decreased cell migration speed which affect the shape of the tumour.

Collagen density within the tumour displays a pattern composed of circles, like a travelling wave. This may be caused by successive cell division: when cells divide, they push each other away, distributing them in circles as the tumour grows rather than uniformly. From the Fig. 6, it is possible to see areas with lower extracellular collagen within the tumour. This pattern does appear clearly in the reference simulation (Fig. 6A i) and in the case with no cell adhesion (Fig. 6A vi). In the case with bi-gel (Fig. 6 A ii), this pattern seems more pronounced in the lower part of the tumour where collagen density is closer to optimal values than in the upper part corrsponding to stiffer matrix. Similarly, the pattern does not seems visible in the case with no oxygen diffusing from the left and right borders of the domain (Fig. 6 A iii). This pattern seems present in the simulation with an heterogeneous matrix (Fig. 6 A v) but it is harder to visually differentiate the wave in this case due to a highly noisy environment. The waves are less visible in both the upper part of the tumour in the case of a bi-gel and in the whole tumour when oxygen diffuses only at the top and the bottom Fig. (6 A iv). In both cases the proliferation of the tumour is reduced which tends to suggest that a slower proliferation may increase the homoneneity of the environment within the tumour. However, migration speed of the cells in these two cases is very different from one another, suggesting that migration speed is not the main factor controlling this pattern. More investigations are needed to confirm what are the factors influencing this pattern.

**Fig. 7:**
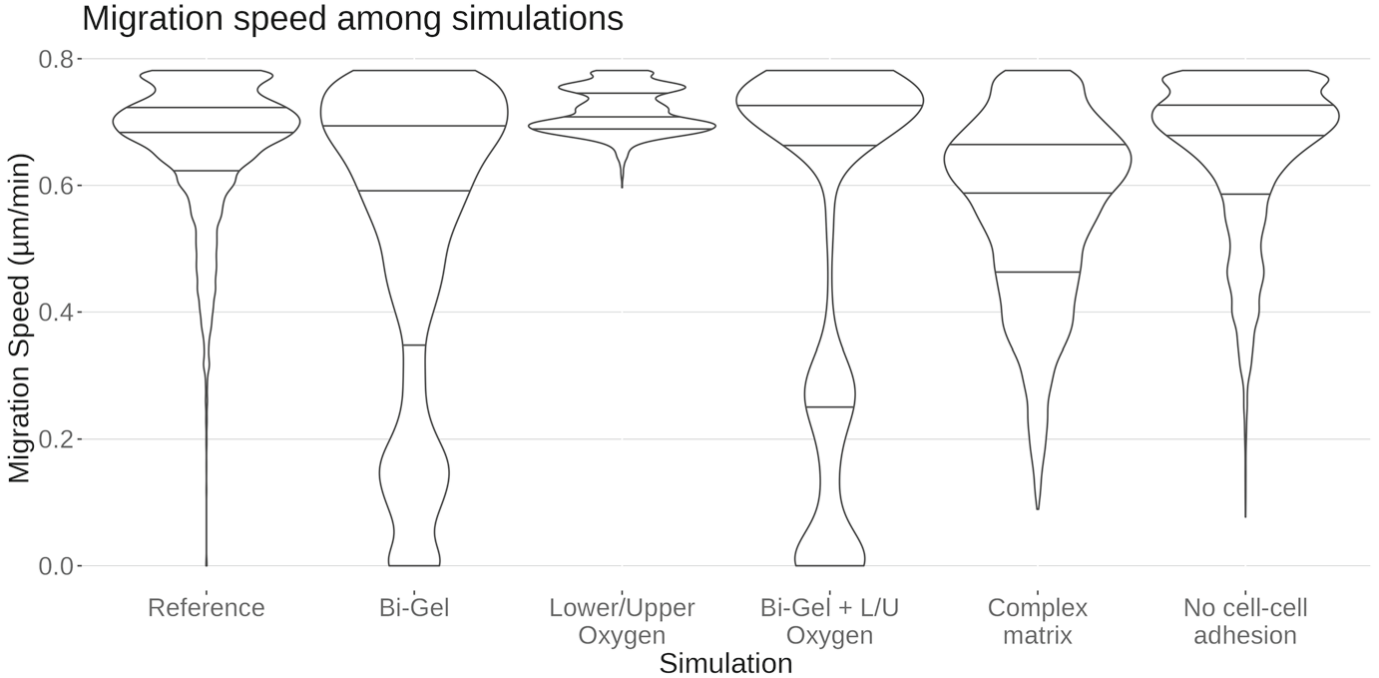
Violin plot of the migration speed in the different collagen conditions

As can be seen in Fig. 7, migration speed in the reference simulation ranges from almost 0 to 0.8 $\upmu$ m/min with most of the values distributed around 0.7 $\upmu$ m/min. Migration was stopped for a few cells in the reference, Bi-Gel and Bi-Gel + Lower/Upper oxygen simulation, as migration speed fall to zero. The simulations where the tumour was grown on a Bi-Gel are the only cases where we can observe two values around which migration speeds seem to aggregate. This is because cells are grown in two different collagen densities depending on whether they are at the top or the bottom of the tumour, enforcing different speeds on the cell. The distribution of migration speed in the complex matrix seems more diverse owing to the random initialization of the matrix. On a homogeneous matrix when oxygen diffuses only at the top and bottom of the domain, migration speed values are comprised between 0.6 and 0.8 which suggests that poorer oxygen conditions strongly select for these values. Probably cells cannot have lower migration speed because they die beforehand due to a lack of nutrients as almost all oxygen is depleted at the end of the simulation.

**Fig. 8:**
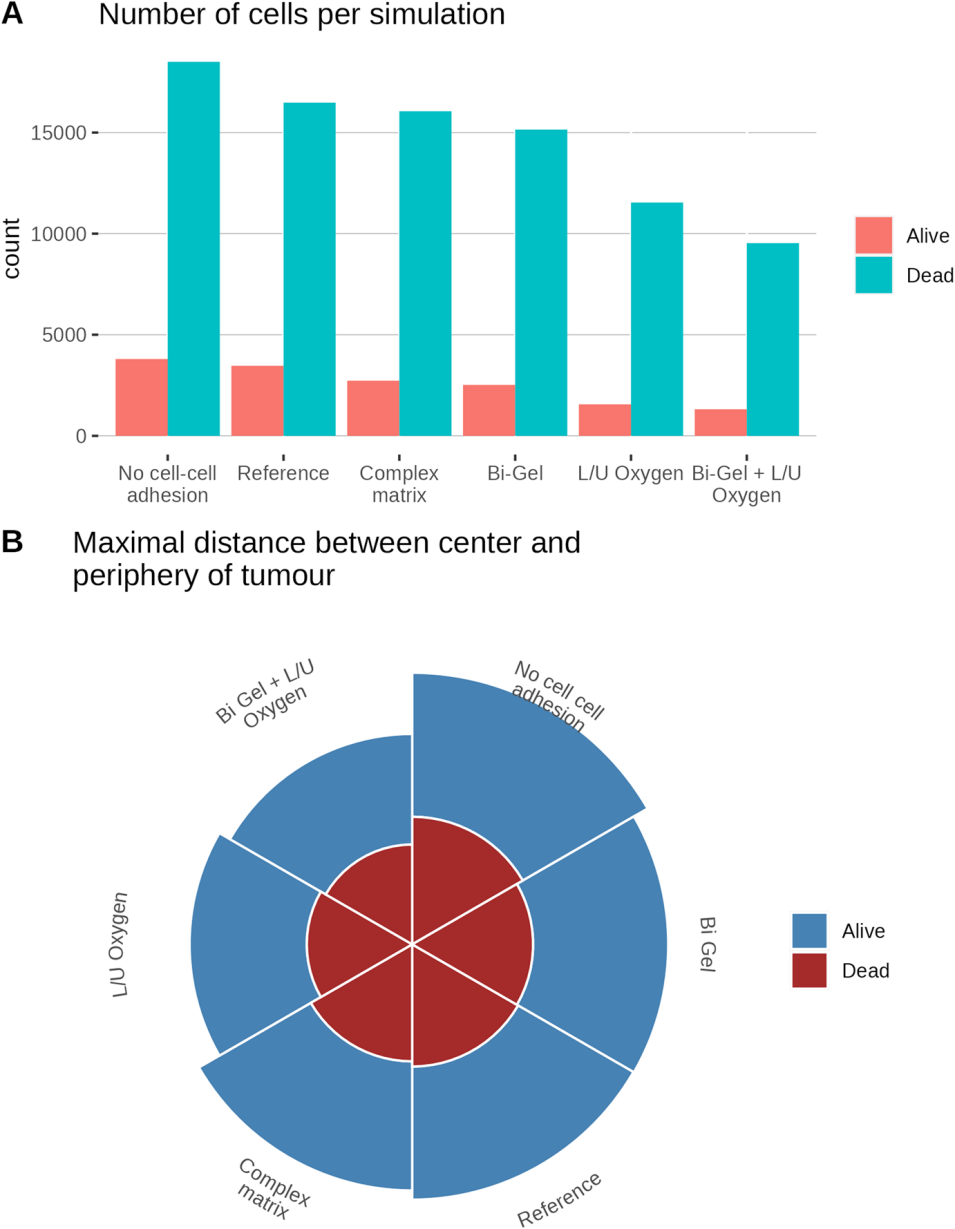
Proliferation of the tumour in the different collagen conditions with (A) a barplot of the number of cells and (B) a pie chart of the maximal proliferation distance. Each slices of the pie chart correspond to a different condition tested, and the height of the slices represent the distance of proliferation which is measured with the radius of the tumour. Hence taller slices represent higher tumour radius. In both cases, the colours represent living and dead cells

The impact of the different conditions on proliferation has been determined by the number of cells (living and dead) at the end of the simulation, and the radius of the tumour (see Fig. 8). The radius of the tumour was calculated by measuring the distance between the centre and the cell the furthest from the center for both the proliferative and the necrotic layers. Tumour growth is maximized in the model when cells have no adhesion and minimized when combining a bi-gel with lower oxygen diffusion. The simulation with no cellular adhesion has the highest number of cells and tumour radius. Taken together, these results show that collagen conditions alone can impact the proliferation of the tumour. Tumour radius and cell number are reduced when oxygen conditions are poorer, showing that oxygen alone can reduce cell proliferation as well. The effect is even more marked when poorer oxygen condition is combined with bi-gel conditions, suggesting a dual effect of both collagen and oxygen.

### Extracelullar Collagen Does not Affect the Appearance of the Warburg Compared to the Genetic Properties of the Cell

The key player of the model in this study is the HIF protein which not only impacts collagen synthesis but the metabolism as well. In previous work Spinicci et al. (2022), we investigated how the interactions between HIF and the metabolic genes LDH and PDH could induce a Warburg Effect. To recall, the Warburg Effect is an increased production of lactate in normoxia. Here we investigated whether extracellular collagen could impact cell metabolism and if conditions that previously triggered the Warburg Effect could lead to the same results. In previous work Spinicci et al. (2022), the Warburg effect could be triggered by reduced HIF degradation by oxygen or through oscillations of oxygen during the simulation. Here we investigated whether this result could be reproduced within the new model. Reduced HIF degradation was simulated by setting $\gamma _{O \rightarrow h} = 17.5$. Oscillating conditions correspond to variations of oxygen boundary conditions: simulation starts at normoxic levels (5 % O_2_ ) and then oxygen levels are slowly decreased over six hours to hypoxic levels (1 % O_2_ ). Oxygen levels are then increased back to normoxia at the same rate and the process is repeated until the end of the simulation.

**Fig. 9:**
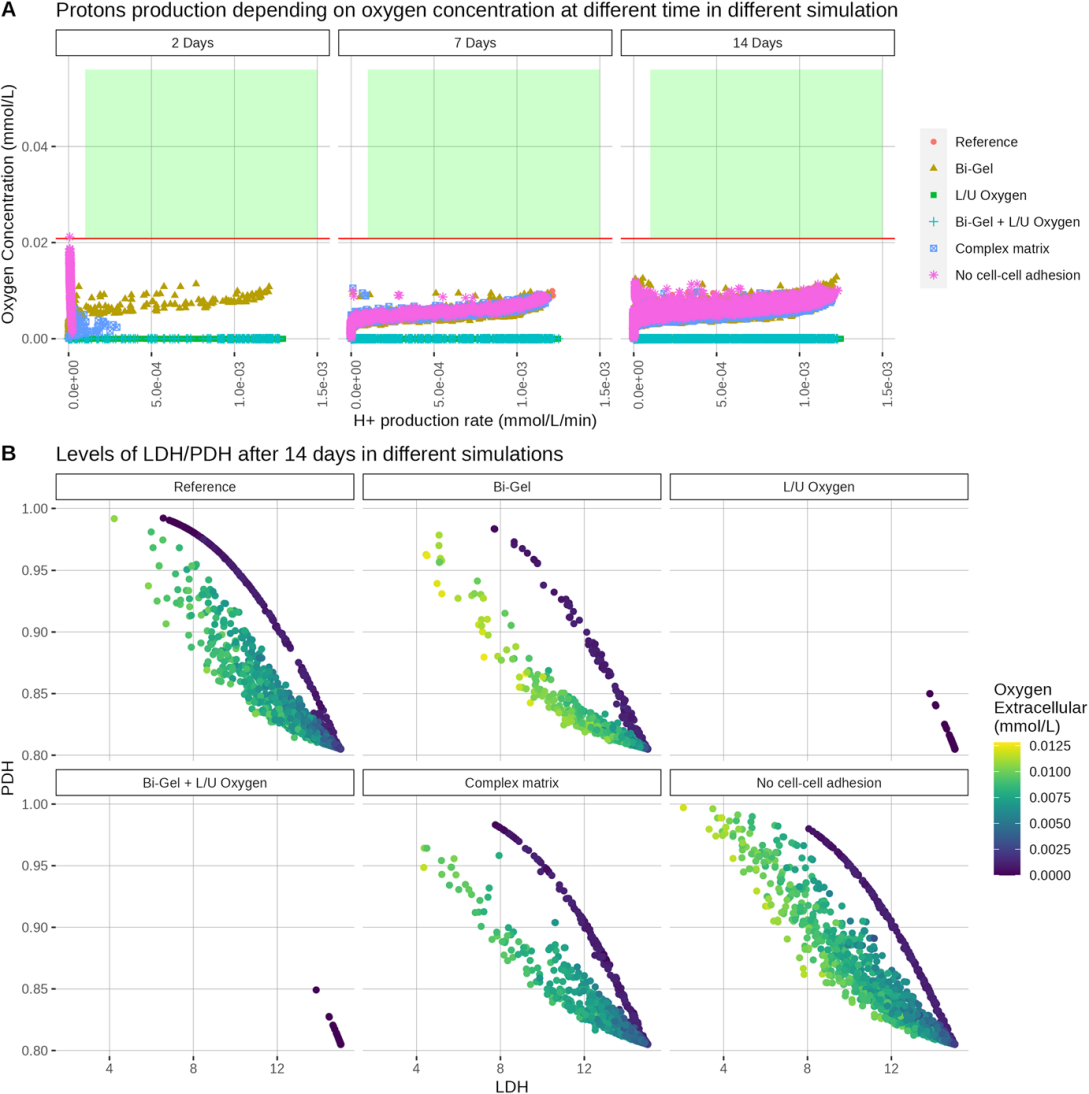
(A) Rate of production of H^+^ at 2, 7 and 14 Days and (B) LDH and PDH levels after 14 days of growth, with different collagen or oxygen settings. In (A), only living cells are represented on the graph, the red line indicates the hypoxia threshold (0.02085 mmol/L or 2% O_2_ ) and the green rectangle represents the region corresponding to a Warburg effect

Figure 9 represents the production of H^+^ (caused by lactate secretion) depending on the extracellular oxygen level in various simulations. As can be seen, extracellular collagen does not seem to impact the Warburg Effect in the model. Results are similar across the simulation in normoxia. Extracellular oxygen levels fall below 2 % O_2_ after only two days and stay below this level in most of the simulation. The lowest values of oxygen levels are reached when the tumour is grown in poorer oxygen conditions. No cells adopted a Warburg phenotype in the Reference, Lower/Upper Oxygen, Complex matrix or Bi-Gel simulations. These results tend to show that collagen has no impact on the Warburg phenotype.

**Fig. 10:**
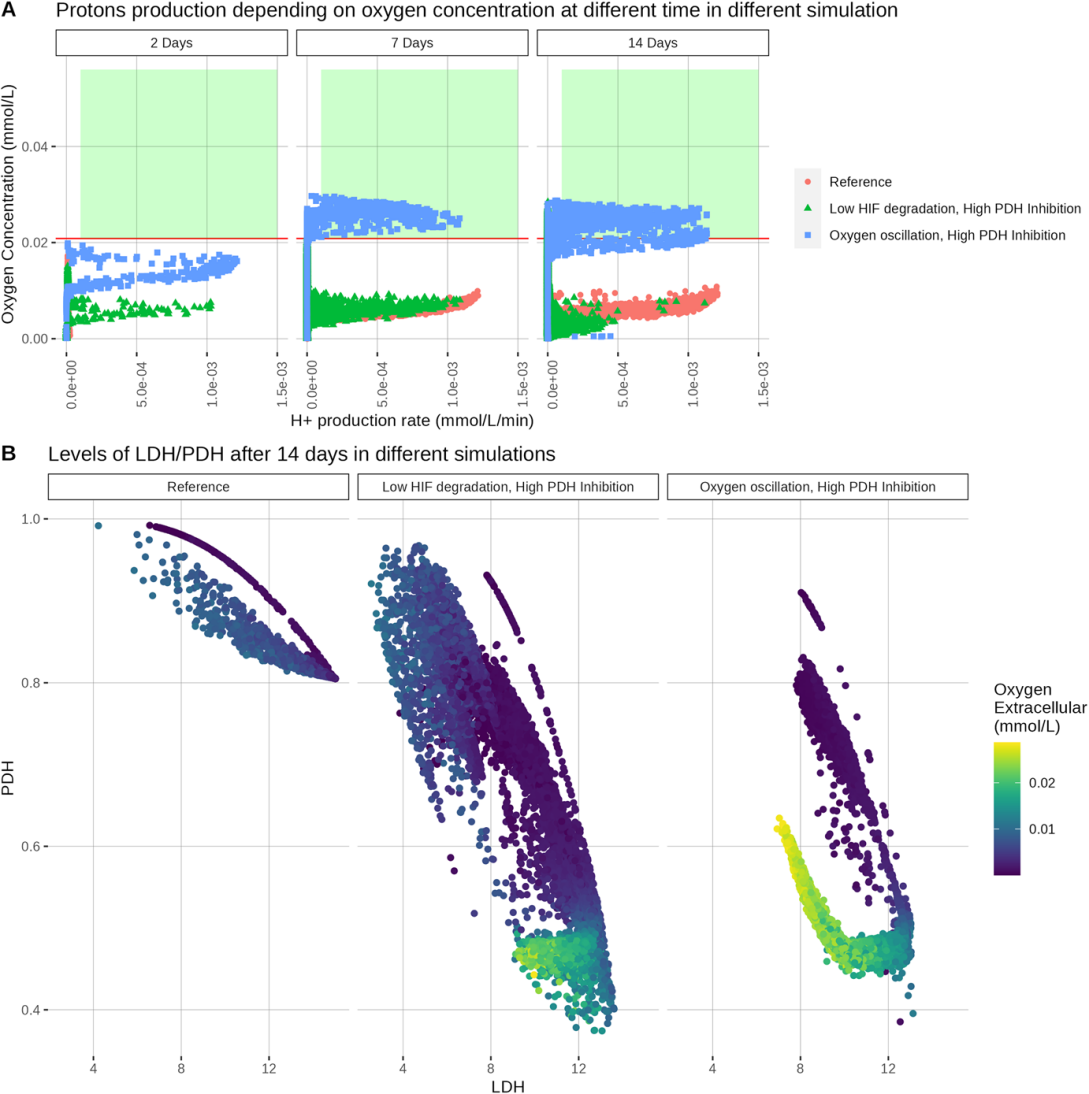
(A) Rate of production of H^+^ at 2, 7 and 14 Days and (B) LDH and PDH levels after 14 days of growth, with different genetic settings. In (A), only living cells are represented on the graph, the red line indicates the hypoxia threshold (0.02085 mmol/L or 2% O_2_ ) and the green rectangle represents the region corresponding to a Warburg effect. Low HIF degradation by oxygen is represented by setting $\gamma _{O \rightarrow h} = 17.5$. High PDH inhibition is simulated by setting $\gamma _{k \rightarrow q} = 0.14$. In oscillating conditions, oxygen concentration is slowly decreased from normoxia (5 % O_2_ ) to severe hypoxia (1 % O_2_ ) during 6h, then oxygen is increased to normoxia at the same rate. This process is repeated until the end of the simulation

As said above, we ran two simulations in the same conditions that triggered the Warburg Effect in previous work but this did not lead to the adoption of the Warburg phenotype by the cell (results not shown). Yet, we noticed that the results of this study are similar to the results obtained in previous work when down-regulation of PDH by pyruvate dehydrogenase kinase (PDK) was decreased. Thus we ran another two simulations with reduced HIF degradation or oxygen oscillation with $\gamma _{k \rightarrow q} = 0.14$ to simulate higher inhibition of PDH by PDK. Figure 10 shows the results when inhibition of PDH is higher. Reducing the downregulation of PDH by HIF (through PDK) rescued the adoption of the Warburg phenotype in conditions with oxygen oscillation but not with reduced HIF degradation. This seems to suggest that environmental conditions can trigger the appearance of the Warburg Effect, yet appropriate genetic parameters are necessary. This would explain why the Warburg phenotype was not observed in the result.

Levels of LDH and PDH, shown in Figs. 9 and 10, seem to support this theory. Three different states can be observed: they correspond to the oxidative state (high PDH/low LDH), the glycolytic state (low PDH/high LDH), and an intermediate state in between the two. This intermediate state is adopted by the cell during its transition from the oxidative to the glycolytic state. LDH/PDH levels follow the same dynamics across the simulations in different collagen conditions. Only the glycolytic cellular state can be observed when oxygen diffuses only at the top and the bottom of the domain, indicating that hypoxia is a strong selector. Lower levels of PDH are attained when increasing the down-regulation of PDH by HIF. This shows that genetic parameters are the main factor affecting the different cellular states in the model. Here, only when oxygen is oscillating and $\gamma _{k \rightarrow q} = 0.14$ we can observe cells in the glycolytic state with normoxic oxygen levels.

## Discussion

In this paper, we studied the impact of the HIF protein on the extracellular matrix through its effect on the genes involved in the modification of collagen. We extended a model of the impact of HIF on the cellular metabolism previously described Spinicci et al. (2022), with matrix remodeling genes. Literature has demonstrated that HIF is able to up-regulate the genes P4HA1, MT1-MMP and LOX. In the model, P4HA1 modulates the secretion of collagen since this protein catalyzes the post-translational modification important for the self-assembly of collagen fibrils into fibers. In contrast, degradation of collagen in the environment is controlled by MT1-MMP which is an enzyme catalyzing the degradation of collagen fibers through cleavage at specific sites. Finally, we describe two states of collagen namely regular collagen and cross-linked collagen. The cross-linking of collagen is controlled by the LOX enzyme which is the main actor catalyzing the reaction resulting in the formation of cross-links between collagen fibers.

Results have shown a relation between the local cellular density and the extracellular collagen in the environment. This further affects the cellular migration speed which is reduced as there is not enough collagen to ensure optimal migration. This is likely to be due to the higher local cellular density causing an increased degradation of the matrix.

As expected, tumour radius in collagen gels with higher collagen concentration was lower as cell migration was slowed down. Simulation in different collagen conditions demonstrated that it affected the shape of the tumour. Unsurprisingly, when tumour was grown in a Bi-Gel where the extracellular collagen at the top was higher than at the bottom of the domain, tumour adopted a “pear” shape. As the upper part of the domain has a higher collagen density, cell migration speed and tumour proliferation were reduced. Yet, when tumour growth is initialized on a complex matrix where extracellular collagen varies on each point of the domain, the tumour forms “branching” at the end of the simulation. Tumour proliferation was reduced when collagen density was not optimal for growth and when oxygen conditions were poorer. Interestingly, when diffusion of oxygen at the top and the bottom of the domain was combined with a Bi-Gel, the number of cells at the end of the simulation was further reduced compared to each condition separately. This tends to show a combined effect of oxygen and collagen on cellular proliferation. Different genetic settings for P4HA1, MT1-MMP and LOX were tested to assess their impact on the tumour growth. Yet results did not change significantly, thus we do not show them in the paper.

The Warburg phenotype has been adopted by any cells in the different collagen conditions simulated. This tends to show that collagen is not relevant to the Warburg Effect in the model. Reduced HIF degradation by oxygen and oscillations of oxygen diffusion did not affect the Warburg Effect either. Adoption of the Warburg phenotype by the cells has been rescued only with oscillations of oxygen when the sensitivity of PDH to HIF was higher. This may suggest that inhibition of oxidative metabolism by HIF is the main factor controlling the appearance of the Warburg Effect in the model. Oscillations of oxygen were necessary to induce the Warburg Effect which means that perturbations of oxygenation may still be required. In this regard, the Warburg Effect is thus a combination of environmental and genetic factors.

In this study, tumour growth was simulated only in 2D to limit the computational cost generated by 3D. However, cell migration differs between 2D and 3D as the cell must overcome different pressures in 3D Lang et al. (2015). While extracellular pH is described in the model, its effect on extracellular matrix is not included here. It has been shown that pH can play a role in promoting cellular invasion Estrella et al. (2013). For example, MMPs undergo acid-induced activation before they degrade the ECM Boedtkjer and Pedersen (2020). Other limitations concern the regulation of MT1-MMP. Firstly, collagen is a molecule able to auto-regulated its own level through a dynamic feedback mechanism that includes regulation of its expression and of the expression of collagen degrading proteinase. At high concentrations, collagen can interact with cell receptors to induce the production of MT1-MMP Tam et al. (2004). Secondly, MT1-MMP self-regulates its activity by cleaving itself, a phenomenon called ectodomain shedding, to remove its catalytic domain which renders MT1-MMP inactive Karagiannis et al. (2004); Itoh and Seiki (2006). Here, the regulation of MT1-MMP only includes the effect of HIF and the auto-regulating mechanisms of collagen and MT2-MMP are not described in the model. However, the effect of collagen up-regulation and MT1-MMP auto-regulation could potentially change the results, especially in high or low collagen concentrations. For example, at low collagen concentrations degradation of collagen could be inhibited in order to favour collagen secretion by the cell.

## Conclusion

In this paper, we investigated the impact of the HIF protein on the remodeling of the extracellular matrix through its effect on the three genes P4HA1, MT1-MMP and LOX. We used a mathematical model that combines genetic regulations, metabolism and collagen processes. The model was implemented in an ABM to accurately describe the local impact by the cell. Results have shown that cells tend to degrade the matrix in the model and that a higher local cellular density leads to a lower extra-cellular collagen density. Areas where the cell motility is hindered show, as would be expected, a shorter distance from the center of the tumour. This impacts proliferation as well since a lower total number of cells is observed at the end of the simulation when collagen density is not optimal. Tumour proliferation was reduced when oxygen diffused only at the top or the bottom of the tumour, and it was maximized when cell-cell adhesion was null. Investigation of the effect of different conditions on the Warburg Effect revealed that adoption of the Warburg phenotype by cells was only observed when the sensitivity of PDH to HIF was increased in combination with oscillations of oxygen diffusion at the border of the domain. This seems to suggest that both appropriate environmental conditions and genetic properties of the cell are required for the appearance of the Warburg Effect.
